# Eleven quick tips to build a usable REST API for life sciences

**DOI:** 10.1371/journal.pcbi.1006542

**Published:** 2018-12-13

**Authors:** Aleksandra Tarkowska, Denise Carvalho-Silva, Charles E. Cook, Edd Turner, Robert D. Finn, Andrew D. Yates

**Affiliations:** 1 European Molecular Biology Laboratory, European Bioinformatics Institute (EMBL-EBI), Wellcome Genome Campus, Hinxton, Cambridge, United Kingdom; 2 Open Targets, Wellcome Genome Campus, Hinxton, Cambridge, United Kingdom; Genome Quebec, CANADA

## Introduction

In recent years, technological advances have greatly expanded the range of data types generated by life sciences researchers. These span domains such as molecular structures, nucleotide and protein sequences, metabolomics, and chemogenomics, resulting in hundreds of public resources holding diverse data sets for reuse in multiple formats [1]. Most resources focus on a specific data type, yet their value for researchers is enhanced once cross-referenced and combined with expert annotation and knowledge. Cross-referencing has increasingly been achieved by implementing website application programming interfaces (web APIs), providing programming-language−agnostic methods to access online resources. Web APIs enable dynamic data exchange between resources, augment websites with additional data, and can provide access to large data sets. Web APIs also enhance adherence to FAIR data principles by making data Findable, Accessible, Interoperable, and Reusable [2], thus increasing the value of those resources.

Representational state transfer (REST) [3] is a popular method for providing interoperability between a client and server [4] using the hypertext transfer protocol (HTTP), the same building block as the world wide web, [5] and a common exchange format, e.g., JavaScript Object Notation (JSON) [6]. REST APIs are considered easier to develop than previous web-service standards, e.g., Simple Object Access Protocol (SOAP). However, REST specifies a set of requirements that any implementation of a REST API must address. Although well-known resources such as the World Wide Web Consortium (W3C) (https://www.w3.org) and the Internet Engineering Task Force (IETF) (https://www.ietf.org/) provide guidance on how to implement such a service, they can be difficult to understand and may have limited documentation. We present here 11 quick tips for creating and maintaining REST web APIs that were developed while implementing various web APIs (https://www.ebi.ac.uk/services) for European Molecular Biology Laboratory, European Bioinformatics Institute (EMBL-EBI)’s data resources.

## Guidelines

### Tip 1. Reduce support costs by providing good documentation

Even the best-designed and most intuitive REST API will languish unused if there is no clear, comprehensive, and up-to-date documentation, preferably including real-world examples. REST API description languages (DLs) document APIs in human and machine-readable formats. A leading DL is the OpenAPI Specification (OAS; originally known as the Swagger Specification, https://swagger.io/), a portable and open specification providing metadata for APIs based on the REST architecture. For example, the OAS is used to generate the interactive documentation of the Proteins API (https://www.ebi.ac.uk/proteins/api/doc/index.html). DLs are not a replacement for prose documentation, walk-throughs of your REST API, and worked examples with detailed request and response specification. DLs can be further enhanced for the life sciences by including references to controlled vocabularies for the returned data formats (e.g., EDAM ontology [7]) that will aid interoperability. Providing all four types of documentation will reduce your support costs.

### Tip 2. Design an API with stable, consistent, and clear URLs

An early first step in building a REST API is to determine the functionalities your web resource intends to provide. REST imposes that every resource should be uniquely addressable and accessed via unique resource locators (URLs) [8], referred to as endpoints. URL schemas that use nouns as labels (which operates with the HTTP verbs [covered in the next tip]) provides a transparent control of actions, for example, to remove a “study” the actions would be DELETE studies/{id}, rather than POST studies/{id}/delete.

Human-readable URLs following this schema style allow easy retrieval of a collection of resources or details of a single entity. The MGnify (formerly called EBI Metagenomics) REST API [9] base URL (https://www.ebi.ac.uk/metagenomics/api/) provides access to several resource collections—such as studies, samples, runs, biomes, and experiment-types—allowing retrieval of over 150,000 publicly available metagenomics and metatranscriptomics data sets, sampled from diverse environments. Endpoints that return multiple entities should provide parameters to help sort and filter returned data. Details about the resource, such as a study, can be retrieved by providing a unique identifier [10] assigned during the archiving process. For example, https://www.ebi.ac.uk/metagenomics/api/latest/studies/PRJEB11419 provides access to The American Gut data sets (https://doi.org/10.1101/277970) and represents the largest human microbiome sample cohort to date. Developing a sensible URL scheme will ensure your REST API is easy to understand and use.

### Tip 3. Use standard HTTP headers to influence how clients will handle your content

A HTTP response from the server to the client is composed of two parts, the information about how the request was processed, including HTTP headers with a status-code, and the message body containing the data from the resource. HTTP headers represent the metadata of a response. Important client-side headers include Origin, used in cross-origin requests (see Tip 6); Accept, used to flag the type of format a client wishes to process (see Tip 5); and Accept-Encoding, used when a client can accept compressed data thereby reducing network traffic. Important server-side response headers include Content-Type, used to state the format of returned data; ETag, used to identify the specific version of the returned content; and Cache-Control, which flags how long a ‘GET’ result can be cached or not cached if using keywords such as No-Cache or No-Store. The latter of the two response headers helps a browser caching the data to reduce traffic between a client and a server.

### Tip 4. Use appropriate standardised data formats

REST APIs can return data in a number of formats, termed media types [11], through a process called content negotiation. The most commonly used media types and formats are:

- application/json (JSON): used to encode nested data structures across multiple languages and is the most prevalent and flexible format;
- application/xml (extensible markup language [XML]): mark-up for nested data with mature tooling for processing;
- text/csv (comma separated values—CSV): a two-dimensional matrix data encoding format;
- application/octet-stream: used when representing any binary data stream;
- text/x-fasta (FASTA): a biological sequence format [12].

**Fig 1:**
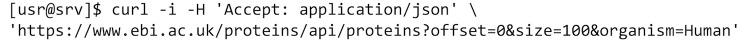
A command line example of the proteins API. A Unix command line using the cURL tool to request information about proteins from the Proteins API in JSON format for human. API, application programming interface; JSON, JavaScript Object Notation.

Life science APIs may wish to support domain-specific formats to help enable tool integration. To begin, a client sends an Accept HTTP header [13] to the server shown on Fig 1, which responds with the best available data representation or an error. The server includes a Content-Type header specifying the format of the returned data.

REST APIs may offer alternative nonstandard ways to configure the format of the data retrieved using file extensions or via a URL parameter, e.g., www.example.com/resource.json or www.example.com/resource?format=json. These alternatives should be avoided if possible, and if not, they should be implemented as an alternative to using the Accept header rather than instead of using the Accept header.

### Tip 5. Use standard HTTP responses to influence how clients will handle your content

**Fig 2:**
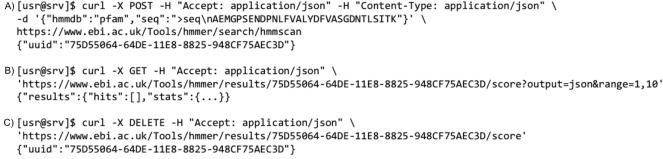
An example of search against HMMER REST API. (A) Search is initialised by POSTing a JSON document with a protein sequence encoded in FASTA format. The client receives a UUID in response. (B) This is used in the second and third queries to retrieve results using GET. (C) The ticket is removed using DELETE. API, application programming interface; JSON, JavaScript Object Notation; REST, representational state transfer; UUID, universally unique identifier.

HTTP defines a set of verbs that can be applied to an endpoint to change the action performed. The most commonly used are ‘GET’ to transfer a current representation of the resource from an endpoint. ‘POST’, ‘PUT’, ‘PATCH’, and ‘DELETE’ (so-called unsafe methods) perform a processing operation, which could be destructive to the data. ‘GET’ must never be used for unsafe operations. ‘POST’ and ‘PATCH’ aside, all other operations are considered idempotent, which means multiple identical requests have the same effect on the state of the resource. For example, the HMMER REST API [14] allows a sequence search against a protein database, as shown in Fig 2.

HTTP also provides five main classes of response status codes:

- 1xx: Informational: request received, continuing process;
- 2xx: Success: the action was successfully received, understood, and accepted, e.g., 200;
- 3xx: Redirection: further action must be taken in order to complete the request, e.g., 302;
- 4xx: Client error: the request contains bad syntax or cannot be fulfilled, e.g., 404;
- 5xx: Server error: the server failed to fulfil an apparently valid request, e.g., 500.

A request to a REST API must return one status code to the client. Incorrect status code usage is misleading and prevents client applications from properly processing a response. For example, HTTP 500 must only be returned when there is a server-side error, not when a client has made a poorly formatted request, in which 400 may be more appropriate.

### Tip 6. Allow your API to be used on other websites by enabling cross-origin resource sharing

All web browsers implement the same-origin policy, a security measure allowing JavaScript code to make requests only between a server and clients of the same origin, which prevents malicious code from hijacking private data such as cookies. Two websites are said to be the same origin if they have an identical scheme (e.g., https), host (e.g., www.ebi.ac.uk), and port (e.g., 443). Cross-origin resource sharing (CORS), part of the Fetch living standard (https://fetch.spec.whatwg.org/), is a technique for circumventing the same origin policy, allowing JavaScript on a web page to consume a REST API served from known and trusted origins securely. The same origin policy is a barrier for open APIs intended to be widely reused.

CORS is automatically used by a browser when a cross-origin request is made. The browser will add an Origin header (whose value is the current origin) to a request. A server may respond with an Access-Control-Allow-Origin header indicating the allowed origin or a ‘*’, indicating that all origins are allowed. Returning the header ‘Access-Control-Allow-Origin: *’ on any ‘GET’, ‘HEAD’, or ‘POST’ request in many cases is sufficient to enable CORS. More complex cases include a preflight step (in which it is evaluated whether an operation can be safely executed) if the request involves a custom header, an HTTP method other than those previously mentioned, or a ‘POST’ request body that is not application/x-www-form-urlencoded, multipart/form-data, or text/plain. For example, a ‘POST’ request in which the posted body is a JSON document is subject to this additional step.

The permissive nature and potential security issues arising from using CORS means that enabling CORS is only recommended for public APIs. CORS plugins exist for all major web frameworks and more information is available from the CORS organisation website (https://enable-cors.org). Additionally, the West-Life consortium has provided a web page that checks CORS compliance (https://www.structuralbiology.eu/network/west-life/creating-web-services).

### Tip 7. Help clients use your API by giving them pregenerated links

**Fig 3:**
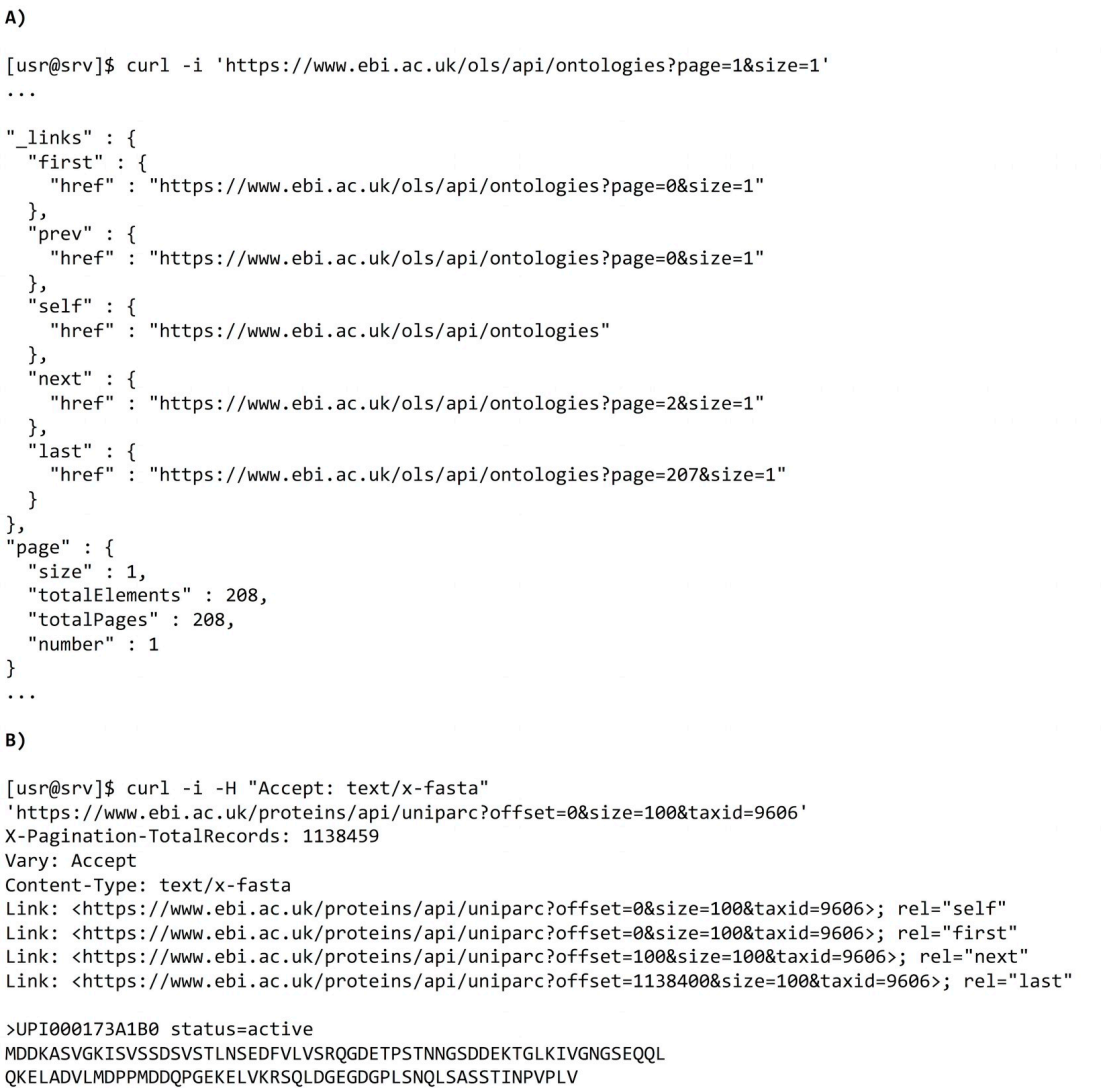
Methods of encoding links into a REST API response. (A) An example query to the Ontology Lookup Service for all available ontologies. The response lists links to access the first, previous, next, and last pages (including supplementary metadata) of the entire collection. (B) An example query to the Proteins REST API to retrieve all sequences in FASTA format. Links are encoded in the Link HTTP response header to be parsed by a client. API, application programming interface; HTTP, hypertext transfer protocol; REST, representational state transfer.

A key concept underpinning the world wide web and HTML is the reconciliation of link navigation by clients. That is, a user clicking on a link requires no knowledge of the target URL; they need only to focus on which link to click. For example, an eCommerce website customer does not need to construct a URL to checkout their purchase. Instead, the website will provide a pregenerated link to click on. Similarly, REST APIs can return actions with a response and inform clients of actions available to them. Providing that these action keywords remain consistent with API changes, a server’s URL scheme is free to change. These APIs are known as being hypermedia driven, which enriches cross-referencing between related resources, a key feature for life science applications. A number of standards exist for creating hypermedia APIs, including JSON for Linked Data (JSON-LD), JSON API, JSON Hypertext Application Language (HAL), and Collection+JSON [15]. A common implementation of this is to paginate through a list of results. For example, the Ontology Lookup Service provides pagination to divide large responses into discrete, manageable chunks [16] using a section of the JSON document called _links listing the functions that can be performed. The URLs provided in the _links section could be external links to other REST APIs or webpages, thereby improving cross-referencing between resources. Where the chosen data format cannot encode these links—as seen, for example, in the Proteins REST API [17] when requesting UniParc data in FASTA format—then HTTP headers can be used as an alternative. Examples of both methods can be seen in Fig 3.

### Tip 8. Authenticate via a standard method

Authentication is the process of identifying a client when the server is provided with a login and password (credentials) that match an authorised individual’s information within an authentication service. We encourage API developers to use common authentication methods such as OAuth 2.0 [18], JsonWebToken [19], and Basic Authentication [20] through an encrypted protocol such as an HTTPS (TLS 1.3) connection and to be aware of security issues that can arise. The Open Web Application Security Project (https://www.owasp.org/) provides extensive information concerning these issues. In addition, you should be aware of the requirements of the General Data Protection Regulation (https://www.eugdpr.org/) if you are processing personal data concerning European Union (EU) citizens.

### Tip 9. Keep your API running at all costs

A useful API is one that remains available at all times. Because APIs are intended for programmatic use, they must scale with demand and be cacheable to avoid reduced network traffic and avoid unintended denial of service attacks. This can be achieved by making your API stateless, i.e., allowing each request to be processed in isolation from others by removing the need for state to be stored on a server. A number of APIs also impose restrictions on access, commonly achieved by giving a quota of requests from a specific internet address over a period of time. APIs offering authentication can allow clients to identify themselves using an API token and have their limits increased or removed. As biological data sets grow in size, with increasingly larger user bases, it may be important to consider horizontal scaling, whereby more servers are added to a pool of servers hosting the API. Because this is primarily a technical solution, hidden behind a load balancer, it will not be discussed further, but software and system architectural design must be considered early during the development phase so an API can scale when under a heavy load. In this situation, a stateless API will be easier to scale than a stateful one. The key is to strike a balance between providing a reliable service and operating within your service constraints.

### Tip 10. Version your API and allow clients to migrate in response to your changes

Versioning is one of the most debated topics among REST API developers and users, with many choosing not to version. A REST API must preserve URL design and data formats to prevent ‘breaking’ client implementations. Resources evolve over time, and these changes necessitate versioning, as epitomised by Roy Fielding who said ‘Versioning an interface is just a “polite” way to kill deployed clients’ [21]. Two approaches are available within the REST guidelines. The first adds a version number in the URL path, making the version visible but affects the stability of future URLs to a resource or endpoint. We recommend the alternative approach, which is to pass a version in the HTTP request header. For example, sending a request to version 3 of the GitHub REST API, whose base URL is https://api.github.com, requires an explicit version set by the header, ‘Accept: application/vnd.github.v3+json’. Extending media types with the vnd prefix (called a vendor-specific media type) is an accepted way to declare multiple schemas of data from a single endpoint, but this also increases API complexity. Providing unversioned shortcuts to the latest version will help drive adoption of an API. Ideally, API clients should be informed of any significant changes to the specification of the URL schema via the documentation and disseminated via social media networks maintained by the service providers.

### Tip 11. Check whether your web framework can help you out

Many of the tips discussed above have already been implemented by a number of REST-compatible frameworks, in a variety of programming languages, e.g., Spring (Java); Django or Flask (Python); Restify, hapiJS, Express, or Loopback (Node.js); and Catalyst or Mojolicious (Perl). If your REST API is built using one of these frameworks, then many of these tips will already be implemented or available through a plugin to simplify implementation.

## Conclusion

The use of simple programmatic methods for making life sciences data available in real time allows researchers to contextualise and interpret their findings against diverse and heterogeneous open data sets, negating costly database replication. Our aim is that life sciences repositories, databases, and archives will provide data managed in adherence to FAIR principles. These 11 tips help us achieve this through API documentation, good API design, reuse of the HTTP standard, and the use of common data formats. REST APIs developed according to the above guidelines allow users to more easily find data, access the content in standards ways, and navigate across complex datasets contained in multiple data resources to address biological questions, thereby maximising knowledge and value of the underlying data. Above, we have presented the guidelines we consider most useful to follow when developing and maintaining REST APIs, and although it is aggregated in the context of the life sciences, we believe this information is of value to any domain.
